# Supplementary material for: Are carbon emissions trading and green financial instruments synergistic? -Comprehensive quantitative research based on content analysis
Source: PLoS One. 2024 Mar 7;19(3):e0298601. doi: 10.1371/journal.pone.0298601 (PMC10919601; doi:10.1371/journal.pone.0298601)
Supplement: S1 Data — (ZIP) [file pone.0298601.s001.zip › Data/地级市Z_F工作报告.zip!/╔┬╬≈/░▓┐╡╩╨/└┤╫╘╣1⁄2╓┌║┼í╛┬φ┐╦╩2╛▌═°í┐_2006.docx]

各位代表：

　　现在，我代表市人民政府，向大会报告“十五”时期经济社会发展情况，并对“十一五”规划和2006年的工作提出建议，请予审议，并请政协委员和其他列席人员提出意见。

　　一、“十五”经济社会发展的回顾

　　撤地设市后的第一届人民政府任期届满。本届政府任期的五年，是我们以科学发展观为指导，紧紧依靠全市人民，扎实落实“十五”计划和一届一次人代会提出的各项任务，全市经济社会发展取得显著成就的五年；是我们积极探索建设绿色安康的发展路子，着力改善基础设施条件，发展壮大绿色产业，奋起改变落后面貌的五年；是我们积极应对和有效处理社会转型期的新矛盾新问题，连续几年战胜严重暴雨洪水灾害，在发展的征程中认真履职的五年。

　　——经济保持较快增长。预计2005年实现生产总值137亿元，比上年增长9.6％，“十五”年均增长8.3%。农业经济结构在市场引导下不断调整。绿色产业、劳务经济和农税改革促进了农村的发展稳定和农民收入的稳步增长。第二产业在调整重组中成长壮大。秦巴医药、汉江水电、绿色食品、安康茧丝、金州矿产等特色工业和建筑房产业呈现较好的发展势头。生态旅游蓬勃兴起，三产服务业快速发展。社会消费品零售总额实现40亿元，比“九五”末增长50.6%。财政收入大幅增长，总收入10.65亿元，支出19亿元，同口径比“九五”末分别增长93.9%、95.8%。支出结构得到优化，多年巨额赤字的局面得以遏制，近两年实现财政收支平衡。

　　——基础建设成效显著。西部大开发战略的实施，有力拉动了我市固定资产投资，累计完成投资232亿元，比“九五”增长1.76倍。西康和襄渝铁路复线全面建设，西康高速全线开工，西汉高速宁陕段加快实施，316高速公路前期工作全面展开，标志着我市大交通体系滞后的瓶颈制约开始破解。二级公路里程达到265公里，新修县际公路430公里,通油路乡镇由76个增加到120个，全市通车总里程由“九五”末5265公里增加到6369公里。喜河电站建设进展顺利，蜀河电站正式开工，330千伏金州和喜河输变电工程即将竣工，城乡电网改造步伐加快，村村通电的目标基本实现。通信网络覆盖率大幅度提高，各类电话用户达到88万户，比“九五”末增长4.3倍。

　　——生态环境明显改善。大力实施生态保护、造林绿化、环境治理三大工程，以汉江为重点的水资源保护、以化龙山为重点的天然林保护、以瀛湖为重点的湿地保护、以秦岭为重点的自然生态保护卓有成效。扎扎实实开展了生态脆弱的汉江两岸、城镇周围、瀛湖周边、交通沿线的工程造林植竹，累计完成退耕还林369万亩、荒山造林156万亩，治理水土流失3750平方公里，依法关闭了43个污染严重的厂矿，新建了一批污水、垃圾处理设施。汉江水质保持在Ⅱ类标准，森林覆盖率由50.2％提高到55％。保护汉江、保护绿色逐步成为全市人民的自觉行动。

　　——城镇建设步伐加快。按照建设山水园林城市的整体构想，修订完善了安康中心城市和各县城的建设规划。全市城镇建设累计投入33.5亿元，比“九五”时期翻了一番。滨江大道（一期）、金州广场、安康师专图书馆和一批高层建筑的竣工，提升了城市品位。市政设施综合改造和交通秩序的整顿治理，改善了城市面貌。县城建设各显特色，新镇新村亮点纷呈，人居环境明显改观，统筹城乡、服务“三农”的功能有所增强。农村人口向非农产业转移12.5万人，全市城镇化率由“九五”末的27％提升到31％，各级政府建设、经营、管理城市的能力有了新的提高。

　　——改革开放逐步深入。完善市场经济体制的各项改革全面展开，农村税费改革成为“大包干”后最受农民欢迎的重大变革，全市取消农业税和除烟叶外的特产税1.61亿元；以产权置换和职工身份转换为重点的国企改革有了较大进展，一批改制后的企业焕发了新的生机。非公有制经济成为经济增长的主导力量。投融资改革有了新的突破，非经营性国有资产管理体制改革激活了政府的资源，打包收购长城资产公司债权为企业解债开辟了新路，与国家开发银行的合作拓宽了融资渠道，财政担保公司的建立有效缓解了部分中小企业贷款难问题。基本完成了农村信用合作社和六县粮食流通体制改革。以治理环境、招商引资、合作交流为重点的对外开放不断扩展，签定招商合同594个，合同引资155亿元，完成投资66.7亿元，引入外资企业23户。安康的知名度和对外开放水平逐步提升。

　　——社会事业全面发展。22个科技项目获得省部级以上奖励，绿色产业建设和工业支柱成长，充分体现了科技进步的巨大作用。10县区全面实现“两基”目标，高考连年取得好成绩，职业教育和成人教育快速发展，安康师专建院升本通过国家考察评估。市县区医院、疾控中心、传染病院（病区）、乡镇卫生院建设力度加大，地方病、传染病防治得到加强，儿童计划免疫率达到91％以上。10县区均被命名为省级计划生育综合服务县，人口继续保持低生育水平。群众性精神文明创建活动扎实推进，“一县一品”文化建设和文化市场整顿取得实效，全民健身运动广泛开展。外事、新闻、档案、方志等部门服务发展的意识进一步增强，气象、地震等工作为防灾减灾做出了积极努力，老龄、慈善、残疾人和妇女儿童等事业取得新的进步。铁路、电力、通信、金融、保险、石油、烟草等行业为地方经济社会发展做出了积极贡献。

　　——人民生活不断改善。经济社会发展使人民群众得到了实惠。城镇居民可支配收入6400元，比“九五”末增加2095元；农民人均纯收入1770元，比“九五”末增加522元；新增城镇住宅面积200万平方米，人均达到26平方米；城乡居民储蓄109亿元，比“九五”末增加57亿元；电视人口覆盖率达到90%。社会保障体系初步建立，城市低保 4.15万人、农村低保 22.65万人，做到了应保尽保。新增就业4万人。城镇职工基本养老、失业、医疗、工伤、生育保险体系进一步完善。对农村计划生育家庭60岁以上老人兑现奖励扶助金735万元。累计投入4.4亿元，实施了469个扶贫重点村建设，扶贫搬迁和生态移民4.9万人，贫困人口减少到68万人。完成防氟改灶9.5万户，修建饮水工程1004处，解决了50万人的饮水困难。为边远村落修建便民桥1034座，改造城镇背街小巷84条，落实了148929名义务教育阶段贫困学生“两免一补”，救助资金2873万元。市县财政筹资偿还了拖欠多年的干部教师工资7583万元。

　　—— 政府建设得到加强。更新施政理念，推进政府机构改革和职能转变，努力为市场主体创造宽松环境。不断完善公共财政体制，着力建设低收入群体的保障机制。自觉接受人大依法监督、政协民主监督、新闻舆论监督和人民群众监督，积极办结人大代表建议和政协委员提案。实行重大事项专家咨询、居民听证和公示制度，政府决策的民主化、科学化水平有了提高。认真开展社会治安综合治理，依法打击各类犯罪活动，强化安全生产监管，高度重视信访工作，基本建立社会利益协调机制、社会矛盾排查调处机制和突发公共事件应急机制，保持了社会大局稳定。切实加强廉政建设，认真纠正损害群众利益的突出问题，严肃查处了一批违法违纪案件，政风建设得到加强，工作作风有了转变，行政效能有了提高。

　　五年来的实践使我们深深体会到：在安康这样一个发展相对滞后的山区市，要实现又快又好的发展，必须坚持科学发展观，立足市情，遵循规律，坚定不移地走具有安康特色的绿色经济之路；必须坚持正确的政绩观，决不能急功近利，更不能无所作为，高度重视关乎安康长远发展的基础建设和绿色资源的积累，让安康的发展经受时间和实践的检验；必须坚持求真务实的作风，崇尚实干，力戒空谈，善于站在前任的肩膀上前进，一张蓝图画到底；必须坚持以人为本，真心诚意地维护和代表人民群众的根本利益，尊重群众的首创精神，尊重基层的辛勤劳动，自觉地植根于人民群众的沃土之中。

　　各位代表！“十五”经济社会发展的成就，是中共安康市委正确领导的结果，是市人大、市政协有效监督、鼎力支持的结果，是全市人民和各条战线团结奋斗、辛勤工作的结果。在此，我代表市人民政府,向全市广大工人、农民、知识分子和干部，向关心和支持政府工作的人大代表、政协委员、各民主党派、人民团体、离退休老同志和社会各界人士，向驻安人民解放军、武警官兵和政法干警，致以崇高的敬意和衷心的感谢！

　　在五年的共同奋斗中，本届政府班子及全体组成人员精诚团结，恪尽职守，扎实工作，为我市的改革发展稳定和政府建设作出了不懈努力，值此，我向退任、离任和转任的各位同仁表示战友的致意！

　　我们还必须清醒地认识到发展中的困难和问题。主要是：农业基础脆弱，工业发展滞后，交通瓶颈制约，人才资金短缺，就业压力增大；非公有制经济规模小，国有企业改革难，财政收支缺口大，一部分城乡低收入群众的生活还很困难；作风不实、落实不力、铺张浪费、损害群众利益的现象在一些地方和部门还很突出；更新思想观念、改善投资环境、转变政府职能的任务还很艰巨。这些都需要我们在前进的道路上，采取积极有效措施，切实加以解决。

　　二、“十一五”规划的目标和任务

　　“十一五”是承前启后的重要时期，我们面临着难得的发展机遇。国家加快实施西部大开发战略，中央转移支付的力度进一步加大，省委、省政府“使陕南成为全省新的经济增长热点地区”和“实现突破发展”决策的实施，为我市加快发展创造了良好的外部环境；三条铁路复线（西安－安康、安康－重庆、安康－武汉）、四条高速公路（西安－安康、安康－陕川界、316国道安康段及西汉高速宁陕段）、四座大型水电站（喜河、蜀河、旬阳、白河）陆续开工建设，不仅从根本上改善安康的基础设施状况，还将拉动我市经济的突破性发展；生态环境建设和“南水北调”中线工程、经济结构调整、消费方式转变，为我们发挥生态优势，做大做强绿色产业，拓展了新的领域和空间；全市人民奋力改变落后面貌、建设美好家园的强烈愿望，必将成为我们加快发展的内在动力。只要我们把握机遇，应对挑战，打牢基础，扎实工作，就一定能够在“十一五”时期实现安康经济社会的突破性发展。

　　根据《中共安康市委关于制定国民经济和社会发展的第十一个五年规划的建议》，市政府组织编制了“十一五”规划，提请这次会议审议。

　　“十一五”发展的基本思路是：坚持以科学发展观统领经济社会发展全局，着力培育以药水游为先导的绿色产业，全力突破基础设施滞后的瓶颈制约，进一步加强生态环境建设与保护，积极推进社会主义新农村建设，实施产业化经营，加速工业化进程，提升城镇化水平，加快发展社会事业，不断改善人民生活，努力构建和谐安康。

　　“十一五”发展的主要目标是：全市生产总值年均增长10%，到2010年，生产总值达到240亿元，人均1000美元以上，从总体上步入小康；全社会固定资产投资累计达450亿元（不含跨市项目），年均增长12%以上；财政总收入达到18亿元，年均增长12%。

　　——社会主义新农村建设初见成效。按照“生产发展、生活宽裕、乡风文明、村容整洁、管理民主”的要求，一乡一两业、一村一两品的产业格局基本形成，农民稳定增收的基础进一步牢固，生产生活条件得到改善，公共服务和民主管理步入规范化轨道。建成一批绿色产业鲜明、基础设施配套、生态环境优美、农家风情浓郁、家庭生活殷实的生态文明新村。

　　——经济结构趋于合理。以药水游为先导的绿色产业体系基本形成，秦巴医药、汉江水电、绿色食品、安康茧丝、金州矿产的规模和市场竞争力显著提高，经济增长方式明显改善。二次产业在生产总值中的比重提升5个百分点，非公有制经济整体实力明显增强。

　　——基础设施建设实现新跨越。铁路、高速公路、民航和地方公路“三横七纵”的大交通格局基本形成，实现出境公路高速化、通县公路二级化、通乡公路油路化、通村公路畅通化。生态环境的质量和效益进一步提高，汉江水质保持Ⅱ类标准，森林覆盖率提高5个百分点，全市经济社会发展开始步入环境友好型、生态效益型轨道。

　　——城镇化进程加快推进。城乡二元结构明显改善，城镇化率达到35%。安康中心城市建设重心北移，江北新区骨架基本形成。以中心城市为龙头，以县城为节点，以特色集镇为基础的城镇格局初步形成，辐射带动经济社会发展和服务“三农”的功能进一步增强。

　　——社会事业全面进步。科技成果转化能力明显增强。义务教育、高等教育、成人教育、职业教育和学前教育的体系更加完善，对农村教育的投入显著增加。公益性的文化事业和经营性的文化产业健康发展。公共卫生防治体系进一步健全，农村新型合作医疗制度全面建立。人口与计划生育的质量得到改善。民主法制建设得到加强，公民素质和城乡文明程度普遍提高。

　　——人民生活明显改善。城镇居民人均可支配收入达到8500元，年均增加450元；农民人均纯收入达到2500元，年均增加150元。基本建立起覆盖城乡的社会保障体系，就业矛盾得到缓解。治安秩序良好，社会稳定和谐。

　　各位代表！未来的五年，我们将实现步入小康的历史性跨越。在新的起点上，安康人民将以自己的勤劳、勇敢、执着与智慧，不惜五年、十年、二十年的不懈追求，向世人展示一幅山川秀美、基础完善、生活宽裕、社会文明的恢弘画卷，真正实现安康大地一派安康！

　　三、2006年工作的任务和重点

　　2006年是“十一五”规划的开局之年。做好新一年的工作意义重大。经济社会发展的目标建议为：生产总值增长10％，固定资产投资超过70亿元，财政总收入增长12％，社会消费品零售额增长13％，城镇居民可支配收入净增300元，农民人均纯收入净增150元，人口自然增长率控制在3.5‰以内，城镇登记失业率控制在4.2％以内。实现上述目标，应重点做好以下工作：

　　（一）做好“三农”工作，建设新农村。推进社会主义新农村建设，是事关发展全局的重大历史任务。坚持“规划先行、分类指导、试点示范、基础突破”的思路，实行群众自主、政府支持，部门帮扶、社会参与，资金捆绑、整村推进的办法，把扶贫开发、移民搬迁、集镇建设、生态旅游结合起来，率先启动100个示范村，有计划、有步骤、有重点地推进具有山区特色的新农村建设。坚持以发展农村经济为中心，以增加农民收入为目标，进一步加大对“三农”的投入，增强农业综合生产能力。稳定发展粮食生产，保持90万吨的总产水平。继续抓好水利与农田基本建设，开工建设汉阴洞河水库，抓紧完善黄石滩水库配套工程，及时整修水毁堤防和病险库塘。解决8万人饮水困难。推进农村电网改造、通信覆盖、广播电视“村村通”。大力推广先进实用技术，高度重视禽流感等疫病防治工作。以强村大户为重点力促畜牧产业的新发展。围绕畜、桑、茶、烟、药等主导产业建设，集中扶持、培育和引进一批实力雄厚、产业化程度高、辐射带动力强的龙头企业。着力培育一批各具特色的专业乡镇、专业村组和专业大户。健全社会化服务体系，积极发展各类合作经济组织，努力提高农业组织化程度。巩固税费改革成果，全面推进以乡镇机构、农村义务教育和县乡财政体制改革为主要内容的农村综合改革。加强农民负担监管，严格执行“一事一议”制度，在民主决策、群众自愿的前提下，引导好保护好农民捐资投劳修路、拉电、引水、兴办教育等公共事业的热情和积极性。

　　（二）狠抓基础项目建设，夯实加快发展平台。突出交通设施和生态环境建设的基础地位。切实加强交通项目建设，全力支持铁路复线、安康铁路枢纽站、高速公路等中省重大交通项目的实施，配合民航实现机场复航。实施“大路带小路”，全面建成平利－镇坪二级公路，新开工西汉高速石泉连接线二级公路，铺设县乡油路300公里，新修通村公路200公里，加强县乡公路和乡村道路养护，有效改善交通状况。加强生态环境建设。以汉江两岸、重点景区、公路沿线、城镇周边生态脆弱区为重点，坚持不懈地抓好退耕还林、荒山造林和公益林建设，完成造林50万亩。组织实施“南水北调”中线工程水源区水保示范县项目。抓好国家级自然生态保护区建设，确定全市原生态河流保护方案。加大污染源监测监控力度，重视生活废弃物的污染治理，坚决关停浪费资源、污染环境的小矿小厂，最大程度地降低环境压力，为可持续发展奠定坚实的基础。

　　（三）壮大特色工业，改善经济结构。实现绿色产业突破性发展，关键在于依托特色资源，培育秦巴医药、汉江水电、绿色食品、安康茧丝、金州矿产五大支柱工业。紧紧围绕深化国企改革、夯实工业基础、打造特色品牌三项重点，切实增强工业经济的整体实力。集中精兵强将，各方配合支持，尽快攻克国企大户改制难点。指导和帮助已改制的企业尽快完成职工身份转换，妥善解决社会保障和失业并轨等遗留问题。大力支持非公有制经济参与国企改革，积极引进大公司大集团，对国有企业实施兼并重组。加快建设水电能源基地，推进汉江干支流开发。喜河电站第一台机组和支流6座电站年内并网发电，加快蜀河电站建设进度，力争旬阳、白河电站前期进点，完成金州和喜河330千伏输变电工程，配套发展符合环保要求的载能工业。大力建设GAP药源基地，提高绿色种养业产业化水平，把农业产业化和工业化有机地统一起来。合理有序地开发矿产资源，加大勘查力度，强化政府调控，在节约资源、保护环境的前提下，发展循环经济，促进集约经营、清洁生产，做强做优矿产建材业。突出安康特色，实施品牌战略，成立品牌建设协调机构，设立专项资金，加强对品牌开发的支持和指导。重点在生物制药和绿色食品上确定一两个骨干产品，加大投入，精心策划，强化营销，做靓品牌，切实发挥扩张效应。推进工业技术进步与创新，抓好一批工业重点技改项目。着力培育、做大做强优势骨干企业，实现工业突破性发展。

　　（四）加强城镇建设，提高管理水平。加快城镇化进程，是改善二元结构、统筹城乡发展的重大举措。从迎接重大发展机遇、实现城区防洪保安的战略高度出发，必须将安康中心城市建设的重心转向江北 。要以实施七里沟大桥、安康大道、滨江大道等重点市政项目为先导，迅速打开江北新区建设的局面，全面启动滨江片区开发，激活生物科技工业园区，改造提升铁路和水电片区，尽快实施一批市政配套项目，同步建设教育、卫生、文化等公共设施，促使人流、物流、资金流转向江北，真正使江北成为我市“十一五”建设投资的热点。严格江南城区建设的规划控制，抓紧城堤公园、金州路、大桥路改造，开工南环东路和香溪大道，力促桥西广场动迁，启动双城广场建设，促进“重心北移，提升江南”布局的实施。进一步加大县城和重点集镇群建设力度，构建服务“三农”、促进产业发展的平台。引导房地产业健康发展，推进经济适用房和廉租房建设，规范物业管理。牢固确立规划的龙头地位，坚决刹住乱批、乱占、乱建，高度重视解决人民群众反映强烈的“脏、乱、差”问题，加强卫生、交通管理，努力改善人居环境，全面提高城市管理水平和文明程度。

　　（五）做大生态旅游，提升三产水平。抓紧完善和颁布实施全市旅游发展总体规划，抓好重点景区景点的控制性详规，依靠规划指导旅游产业的发展。深化旅游体制改革，建立景区景点国家所有、行业管理、企业经营的运行机制，将开发前景广阔的景区景点推向市场，吸引大公司大集团投资开发，努力实现旅游业由政府推动为主向企业运作为主的转变。着力打造瀛湖旅游品牌。在完成瀛湖核心景区造林任务的基础上，扎实推进77平方公里环湖坡面的工程造林，力争通过五年努力，将瀛湖库区建成生态环境示范区、特色产业精品园、汉水风情观光带和高品位“农家乐”集群，让瀛湖山明水秀，使之真正成为大西北的水上胜景。加快香溪洞、南宫山、神河源、千家坪、云雾山、天华山、朝阳沟等景区的深度开发。打造安康至岚皋休闲度假游、安康至紫阳民俗风情游、安康至平利田园风光游三条精品线路。优先安排连接景区景点的交通项目，完善旅游配套服务设施，加强旅行社的管理和引导，规范景区景点等级评定，积极构建和申报国家高等级景区。加大宣传促销力度，继续办好一年一度的汉江龙舟节。认真研究我市旅游市场的季节性特征，有针对性地抓好淡季市场开发，促进旅游业持续均衡发展。依托旅游产业发展，运用现代经营方式提升改造宾馆饭店、餐饮娱乐、交通运输 、商品零售等传统服务业，促进电子商务、物流配送、金融保险、信息和法律服务等现代服务业快速成长。认真研究消费趋势和消费热点，促进消费结构升级。加快农村集贸市场建设，促进农产品流通，加强农村消费市场监管，不断改善和努力扩大农村消费。

　　（六）着力推进改革创新，放手发展非公经济。在深化国企改革、农村综合改革的同时，着力推进重点领域、重点行业的改革。以政府资产管理体制改革为重点，全面推行非经营性国有资产的统一管理，严格实行政府垄断土地一级市场，真正行使国家对矿山、河流等重要资源的所有权；以完善流通体制为重点，深化商贸、粮食流通体制改革，加快建设批发市场、区域市场和产地市场，促进商品集散交易；以理顺财政管理体制为重点，进一步完善财政支出结构，努力缓解县乡财政困难；以科技创新为重点，整合科研力量，组织对新产品研发、特色资源开发与保护等方面的联合攻关，支持科技人员开展技术承包、领办科技项目；继续把改善投资环境作为“生命线”工程来抓，把优势资源、重点产品、开发项目推向市场，完善和创新招商引资方式，力争当年引资20亿元。

　　认真落实国务院、省政府关于支持非公有制经济发展的政策措施。按照“平等待遇，开放领域，引导产业，调整结构，创新服务”的基本思路，加强和改进政府监督、管理和服务，消除各种体制性障碍，吸引民营资本投向工业，参与国企改革，投资公用行业、基础设施、中介服务和教育文化卫生等领域。引导非公有制企业按照现代企业制度的要求，完善法人治理结构，创新体制机制，促进技术进步和产业升级。充分发挥市贷款担保公司的作用，切实帮助民营企业缓解融资困难，促进非公有制经济健康发展。

　　（七）大力发展社会事业，推动社会全面进步。高度认识教育对经济的奠基和拉动作用。强化政府对义务教育的保障责任，努力增加教育投入。抓好农村寄宿制学校、中小学危房改造和现代远程教育。巩固“两基”成果，扩大高中办学规模，加快发展职业技术教育，放手发展民办教育。全力争取安康师专建院升本成功。整合市区教育资源，优化安康城区教育布局，满足社会对教育的需求。大力发展公益性文化事业，加强县、乡镇、村文化设施建设。积极发展经营性文化产业，切实加强文化市场的培育和管理。深入开展群众性的精神文明创建活动。进一步健全疾病防治体系和突发卫生事件应急机制。深入开展爱国卫生运动。切实加强高致病性禽流感、艾滋病、结核病、肝炎等重大传染疾病和地方病的预防控制。继续强化儿童计划免疫。抓好农村卫生设施建设，完成岚皋、旬阳、紫阳三县乡镇卫生院改扩建和汉滨区艾滋病防治示范区卫生院建设任务。加强医德医风建设，严格医疗服务监管，遏制重大医疗事故发生。强化对药品招标采购的监督，高度重视解决群众看病难看病贵的问题。广泛开展全民健身运动，不断提高人民群众的健康素质。坚持计划生育一把手负责、一票否决和黄牌警告制。夯实基础工作，构建规范化、法制化的长效管理机制。认真兑现农村部分计划生育家庭奖励扶助政策。有效治理出生人口性别比偏高问题，稳定低生育水平，推进优生优育，提高人口素质。

　　（八）维护群众利益，促进社会和谐。解决好人民群众最关心的就业、社会保障、上学看病和社会稳定等问题，是实现社会和谐的基本要求。要大力发展就业容量大的劳动密集型产业和企业，积极开发公益性就业岗位，优先安置失业人员尤其是“4050”人员。探索安置复员退伍军人的新途径，引导大中专毕业生面向基层就业、依靠市场就业和自主创业。认真落实就业再就业的税费优惠政策。对职工再就业和农村转移的劳动力予以免费培训。全年城镇新增就业1.1万人，劳务输出稳定在40万人的规模，其中有组织的输出达到15万人。

　　在巩固完善城乡特困人口最低生活保障制度的同时，提高城市低保人口中60岁以上老人的补助标准，并从今冬开始，每户增加四个月每月50元的取暖补助。对城乡低保家庭子女考入大中专院校的给予救助。财政预算中每年安排300万元，专项用于背街小巷的改造及环卫保洁，下工夫解决路不平、灯不明、厕所脏的问题。按既定的项目和投资，继续抓好边远村落便民桥建设。努力缩小城乡居民在医疗保障和义务教育方面的差距。从2006年开始，对全市农村义务教育阶段的学生免收杂费，对贫困家庭学生提供免费课本和寄宿生活费补贴，对城市低保家庭学生实行与农村学生一样的免杂费政策。在巩固旬阳县试点成果的基础上，扩大实施平利、白河、石泉三县新型农村合作医疗，用三年时间，将全市250万农民全部纳入新型农村合作医疗范围。真正让城乡群众共享改革发展成果。

　　高度重视安全生产，建立和完善安全生产长效管理机制，坚持“四不放过”原则，坚决遏制重特大事故发生。完善以洪水和山地灾害为重点的防灾减灾预案，最大限度地规避和减轻灾害损失。建立健全突发公共事件应急处理机制。加大保健品、药品和餐饮业市场监管力度，保障群众健康安全。正确认识和处理新形势下的人民内部矛盾，加强人民调解和信访工作，及时将苗头性、倾向性问题化解在基层和萌芽状态。进一步加强民族宗教工作。认真实施“五五普法”，积极创建“平安安康”。切实落实社会治安综合治理措施，高度重视未成年人的教育和保护，及时侦破大案要案，依法打击抢夺抢劫、入室盗窃、敲诈勒索等犯罪，坚决铲除黑恶势力，惩治街痞村霸市霸，为人民群众创造安居乐业的社会环境。

　　 四、切实加强政府能力建设

　　当前我们正处在一个夯实基础、酝酿突破的关键时期，高度重视和切实加强政府的能力建设，就更为紧迫和重要。

　　增强服务发展的能力。在市场经济条件下，政府组织经济社会发展的作为，主要体现在服务能力上。要增强激发市场主体活力的能力，切实解决政府职能的越位、缺位和错位问题，真正把精力转向经济调节、市场监管、社会管理和公共服务上，引导生产要素的合理流动和优化配置，通过创造条件，改善服务，协调矛盾，让各类市场主体的创造活力竞相迸发；要增强推进体制创新的能力，指导国有企业改革攻坚，放胆发展非公有制经济，敢于冲破旧体制的束缚，着力创新促进发展的体制机制；要增强对外开放的能力，努力创造公正透明的政策环境、高效优质的服务环境、平等竞争的市场环境、公开公正的法制环境、诚实守信的社会环境，让投资者放心、安心、舒心，搭建开放、开发、发展的广阔平台；要增强服务“三农”的能力，始终把农村、农业、农民问题放在全局的首位，引导全社会共同参与，动员各条战线出力献策，使社会主义新农村建设成为全市上下的共同行动。

　　增强依法行政的能力。市场经济是法制经济，政府必须成为法制政府。政府工作人员必须熟悉法律、运用法律、遵守法律，努力提高依法行政的本领。依法行政首先要增强法律意识，下功夫转变普遍存在的不学法不懂法的状况，把学习法律作为能力建设的必修课。要善于运用法律，改变习惯于靠行政手段、凭经验办事的方式，在法律的范围内行使职权，用法律规范政府行为，依靠法律调处社会矛盾，运用法律维护市场秩序。要自觉遵纪守法，坚决维护《宪法》和法律的权威，自觉接受人民代表大会及其常委会的法律监督、人民政协的民主监督、舆论监督和公众监督，建立健全民主科学决策的程序和机制，建立完善教育、制度、监督并重的惩治和预防腐败体系，认真执行《行政许可法》和《公务员法》，依法管理公务员队伍，确保人民的权力真正服务于人民。

　　增强协调利益的能力。妥善协调各方面的利益关系，是政府工作的重要内容，是我们必须具备的基本功。要增强处理人民内部矛盾的能力。深入研究和把握新形势下群众工作的特点和规律，针对人民群众思想观念、利益需求、生活方式的新变化，加强信访工作，畅通诉求渠道，完善社会利益协调和社会纠纷调处机制，倡导人与人和谐相处，及时把矛盾化解在基层和萌芽状态。要增强处理不同利益群体之间关系的能力。提高在各种利益交织碰撞的复杂情况下驾驭局面、协调关系、调节利益的本领。在经济发展、项目建设、征地拆迁、国企改革等具体问题的处理上，尤其要正确认识和妥善处理长远利益与眼前利益、全局利益与局部利益、根本利益与个人利益的关系，合理调节利益群体之间、社会成员之间的利益差别，兼顾各种合理的利益诉求，使各类利益主体各得其所。要增强维护社会公平与正义的能力。把政府的公共资源，更多地向社会事业、社会保障、基础设施和“三农”方面倾斜；把政府的注意力，更多地放在维护弱势群体的利益上；把政府的工作职责，更多地放到解决群众生产生活中最紧迫的实际问题上。

　　增强组织落实的能力。落实是为政之要，不抓落实、不会落实的政府是无能的政府。增强组织落实的能力，最重要的就是全市上下牢固树立以经济建设为中心的思想，一切工作都要服从和服务于经济建设这个中心，要用发展凝聚人心，调动全社会的一切积极因素，形成一心一意搞建设、齐心协力谋发展的强大合力。增强组织落实的能力，最关键的就是确立正确的导向。要坚持正确的用人导向，激活干部队伍的发展热情和创造活力，真正做到主战场出干部、凭实绩用干部、抓落实看干部，决不能让那些埋头苦干、任劳任怨、政绩突出而又不事张扬的人吃亏；要坚持正确的政绩导向，切实解决干与不干、干多干少、干好干坏一个样的问题，坚决纠正无所事事、急功近利、讨价还价的不良倾向，建立和完善科学合理的目标责任、工作评价、政绩考核制度。增强组织落实能力，最要害的就是大兴求真务实之风。切实转变领导作风、干部作风和工作作风，克服不切实际的主观主义，华而不实的形式主义和脱离群众的官僚主义，善于谋事，敢于碰硬，勇于奉献，认准的事情扭住不放，决定的工作一抓到底，确定的任务严格奖惩，下力整治软懒散，坚决精减会议文件，切实改进文风政风，加强部门间的协作配合，确保政令畅通和高效运转。

　　团结是我们战胜困难、不断前进的法宝，团结是力量之源、胜利之本，团结是294万人民的福祉，维护团结就是维护294万人民的根本利益。面对新形势新任务，我们要像爱护自己的眼睛一样，倍加珍惜团结稳定的大好局面。让我们都用自己的一片真诚、无私胸襟，为了成就绿色安康的建设大业，肩并着肩、手挽着手，共创美好的明天！

　　各位代表！新的形势激励着我们，新的任务召唤着我们。让我们站在新的历史起点上，用科学发展观统揽全局，以奋发有为的精神状态迎接“十一五”的开局之年，把建设繁荣文明和谐新安康的宏伟事业不断推向前进！
